# Biomechanical study of C1 posterior arch crossing screw and C2 lamina screw fixations for atlantoaxial joint instability

**DOI:** 10.1186/s13018-020-01609-6

**Published:** 2020-04-17

**Authors:** Chuang Liu, Allieu Kamara, Yunhui Yan

**Affiliations:** 1 State Key Laboratory of Mechanical Behavior and System Safety of Traffic Engineering Structures, Shijiazhuang Tiedao University, Shijiazhuang, 050000 Hebei People’s Republic of China; 2 Department of Pediatric Orthopedics, Shengjing Hospital of China Medical University, Shenyang, 110004 Liaoning People’s Republic of China; 3 School of Mechanical Engineering & Automation, Northeastern University, Shenyang, 110819 Liaoning People’s Republic of China

**Keywords:** Biomechanics, Atlantoaxial instability, Finite element, Range of motion, Stress

## Abstract

**Background:**

The biomechanics of C1 posterior arch screw and C2 vertebral lamina screw techniques has not been well studied, and the biomechanical performance of the constructs cannot be explained only by cadaver testing.

**Methods:**

From computed tomography images, a nonlinear intact three-dimensional C1-2 finite element model was developed and validated. And on this basis, models for the odontoid fractures and the three posterior internal fixation techniques were developed. The range of motion (ROM) and stress distribution of the implants were analyzed and compared under flexion, extension, lateral bending, and axial rotation.

**Results:**

All three kinds of fixation techniques completely restricted the range of motion (ROM) at the C1-2 operative level. The C1-2 pedicle screw fixation technique showed lower and stable stress peak on implants. The C1 posterior arch screw + C2 pedicle screw and C1 pedicle screw + C2 lamina screw fixation techniques showed higher stress peaks on implants in extension, lateral bending, and axial rotation.

**Conclusions:**

As asymmetrical fixations, C1 posterior arch screw + C2 pedicle screw and C1 pedicle screw + C2 lamina screw fixations may offer better stability in lateral bending and axial rotation, but symmetrical fixation C1-2 pedicle screw can put the implants in a position of mechanical advantage.

## Background

Odontoid fracture with atlantoaxial dislocation is a serious injury impacting on the stability of the atlantoaxial joint. Atlantoaxial instability may result in disabling pain, cranial nerve dysfunction, paresis, and even sudden death [1]. Most of the odontoid fracture associated with atlantoaxial dislocation requires surgical intervention. Surgical fixation of atlantoaxial instability is a challenging procedure due to the complex anatomy, special physiological motion, and individual variation. Currently, posterior screw-rod fixation has been used widely in upper cervical spine fusions. The main reason is that it can provide excellent biomechanical stability when compared with Gallie wires, Brooks wires, Halifax interlaminar clamps, and Apofix laminar hooks [2–5]. Gradually, the transpedicular screw technique has been regarded as the gold standard for spine fusion. However, C1 pedicle screws need to go through the vertebral artery groove, which may place the vertebral artery (VA) at risk, while C2 pedicle screws often face difficulty in screw placement due to the variable locations of foramen transversarium. This results in a rate of vertebral artery injury that can be as high as 20% [6, 7].

To address these concerns, C1 posterior arch screw (C1PAS) fixation and C2 lamina screw (C2LS) fixation have been emerging as a useful stabilization technique in recent years. In 2000, Floyd and Grob [8] first used C1 posterior arch screws to fix grafts during C1-2 fusion in five patients and achieved good clinical results. Donnellan et al. [9] used these techniques when the posterior C1 arch was deficient and obtained ideal postsurgical outcomes. In 2013, Guo-Xin et al. [10] designed C1 posterior arch crossing screw fixation both to avoid disturbing the heads and to increase the lengths of the screws. They studied its feasibility on the basis of anatomical measurements and in vitro biomechanical testing. As for C2 fixation, Wright [5] first proposed the use of C2 translaminar screws, which was shown to provide equivalent stability to C1 lateral mass screws and C2 pars screws by biomechanical analysis [11–13]. Some in vitro testing and short-term follow-up studies showed that the C2 laminar screw fixation provides equal stiffness to the pedicle screw fixation [12, 14, 15]. In addition, these constructs posed no risk of injury to the vertebral artery (VA) and were further shown to be a good alternative in clinical practice.

To date, cadaveric biomechanical studies of C1 posterior arch screw and C2 vertebral lamina screw fixations have been performed, but the biomechanical performance of the constructs cannot be well studied only by the cadaver testing. The clinical application of both techniques was hindered all along due to a lack of precise theoretical analysis. The purpose of this study was to investigate the biomechanical properties of C1 posterior arch screw and C2 vertebral lamina screw fixations for the atlantoaxial instability to compare their stability and stress distribution of the implants against that of pedicle screw fixation.

## Methods

### Establishment and validation of finite element model of normal atlantoaxial vertebra

**Fig. 1:**
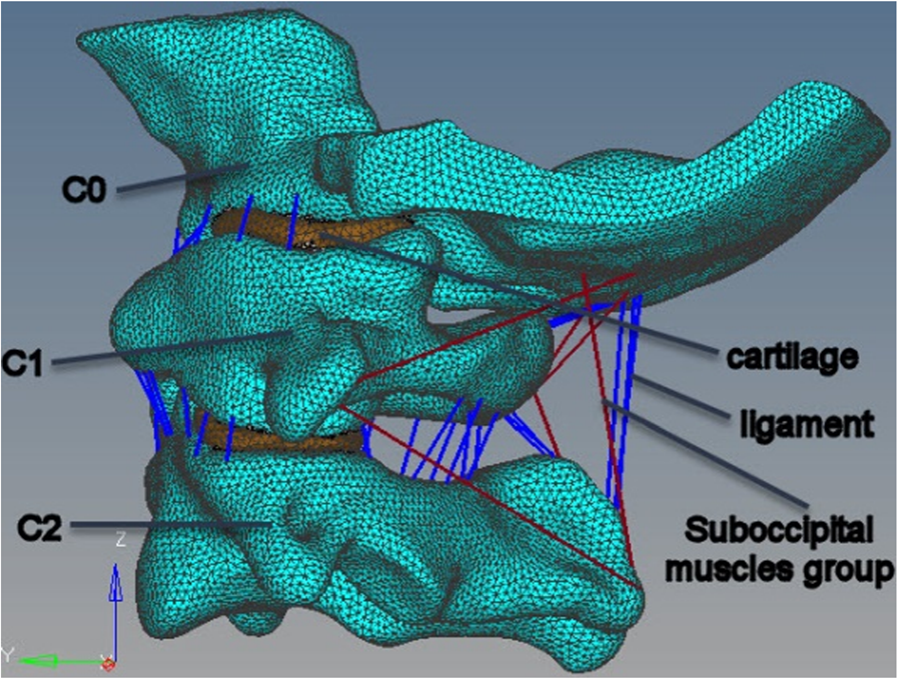
Finite element model of atlantoaxial

This study was supported by Sheng Jing Hospital affiliated to China Medical University. A detailed, anatomically accurate three-dimensional finite element model (FEM) of the atlantoaxial vertebra was developed based on CT images of a healthy 25-year-old male volunteer (weight 70 kg, height 175 cm) by simpleware 6.0. The vertebral geometry data for the atlantoaxial vertebrae were obtained from the 0.5-mm-thickness CT scans (transverse slices). In brief, the established occipital-atlanto-axial complex shown in Fig. 1 contained the following major components: the lower part of the occipital (C0), atlas (C1), axis (C2), and intervertebral cartilage; eight key ligaments—transverse ligament (TL), anterior and posterior longitudinal ligaments (ALL and PLL), capsular ligament (CL), flavum ligament (LF), apical ligament of dens (LAD), alar ligament (AL), and membranae tectoria (MT); and suboccipital muscle group, including rectus capitis posterior (RCP) major and RCP minor muscles, musculus obliquus capitis superior, and musculus obliquus capitis inferior. The vertebral bodies were composed of a cancellous bone core and a 1-mm-thick cortical mask. All ligaments were subjected to only tension and were therefore set as “no compression” in hypermesh during meshing, to ensure that all the ligaments produce force when pulled. The material parameters and the type and number of elements for each part are summarized in Table 1 [7–9].

**Table 1:** Material property, designations, and element number of the finite element model

Component	Element type	*E* (MPa)	*ʋ*	Cross-sectional area (mm)	Element number
Cortical	C3D15, C3D10M	1200	0.29	–	187865
Cancellous	C3D10M	450	0.29	–	132759
Cartilago articularis	C3D10M	10	0.3	–	11343
TL	S8R	20	0.3	–	114
ALL	T3D2	54.5	0.3	6.1	6
PLL	T3D2	20	0.3	5.4	6
CL	T3D2	20	0.3	46.6	25
LF	T3D2	1.5	0.3	50.1	5
LAD	T3D2	10	0.3	5.0	3
AL	T3D2	7	0.3	22.0	2
MT	T3D2	10	0.3	6.0	5
Suboccipital group	T3D2	150	0.2	–	8
Implant	C3D8	110,000	0.33		–

To validate our model, we compared the range of motion (ROM) of the C0-1 and C1-2 segments of the intact FEM with the results of the cadaveric experiment by Panjabi et al. [10] and the upper cervical finite element analysis by Brolin et al. [5, 11], under the same boundary and loading conditions. Using the principle of Panjabi’s biplanar stereoscopic technique, the ROM of the atlantoaxial segments was calculated based on nodal displacement. Our results showed reasonable agreement with that of Panjabi’s cadaveric experiment and Karin Brolin and Zhang’s finite element study, as presented in Table 2.

**Table 2:** Predicted ROMs under different physiological conditions compared with other studies

Load	Segments	Panjabi 1991	Karin Brolin 2004	Hao Zhang 2007	This study
Flexion	C0-1	14.4 ± 3.2	18.2	14.5	8.34
	C1-2	12.7 ± 3.2	11.3	15.0	11.2
Extension	C0-1	14.4 ± 3.2	10.5	13.3	8.69
	C1-2	10.5 ± 5.0	14.0	12.7	7.33
Lateral bending	C0-1	5.6 ± 3.0	3.0	5.5	3.61
	C1-2	12.6 ± 7.0	4.0	5.9	5.17
Axial rotation	C0-1	3.3 ± 2.3	6.1	8.5	4.7
	C1-2	37.4 ± 9.0	23.3	30.6	28.34

### Static analysis of the three kinds of fixations

**Fig. 2:**
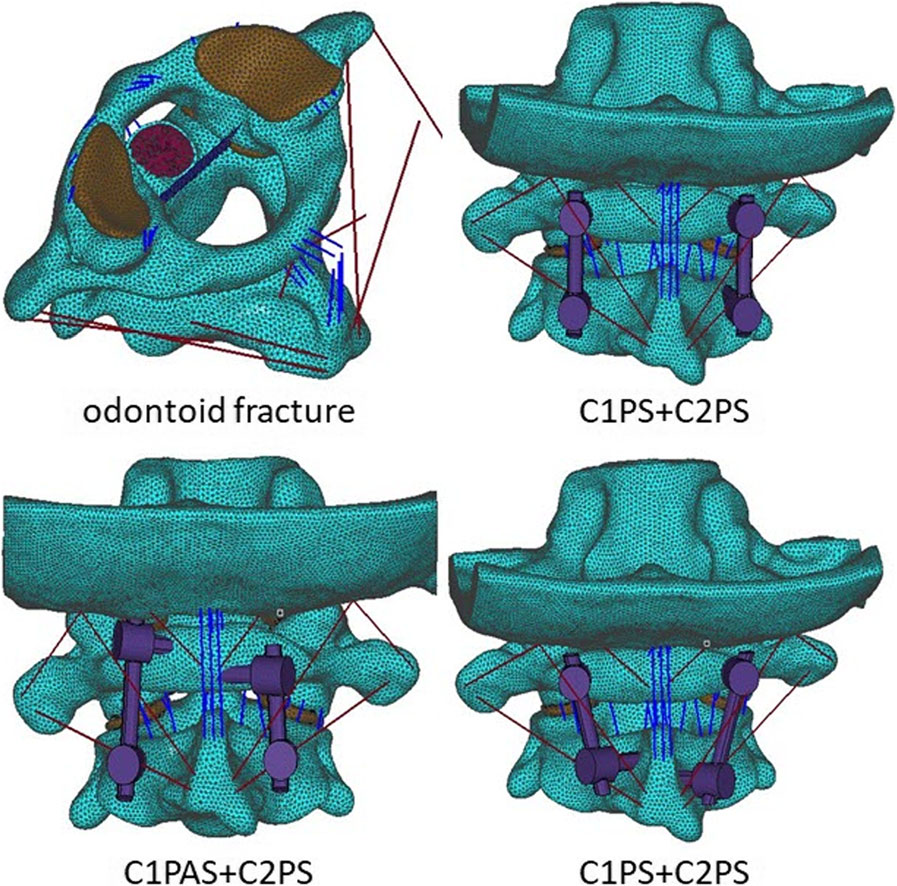
Odontoid fractures and three fixation models of atlantoaxial

The FEM of intact atlantoaxial vertebrae was employed to develop the model of odontoid fracture. Screw-rod fixator models were created based on the universal cervical polyaxial screw (3.5 mm diameter, 24 mm length) manufactured by Shandong Weigao Orthopedic Materials Co. Ltd. The internal fixators were implanted into the odontoid fracture model with the conventional surgical approach using the three techniques: C1-2 pedicle screws (C1PS + C2PS), C1 posterior arch screw + C2 pedicle screw (ClPAS + C2PS), and C1 pedicle screw + C2 lamina screw (C1PS + C2LS), as shown in Fig. 2.

**Fig. 3:**
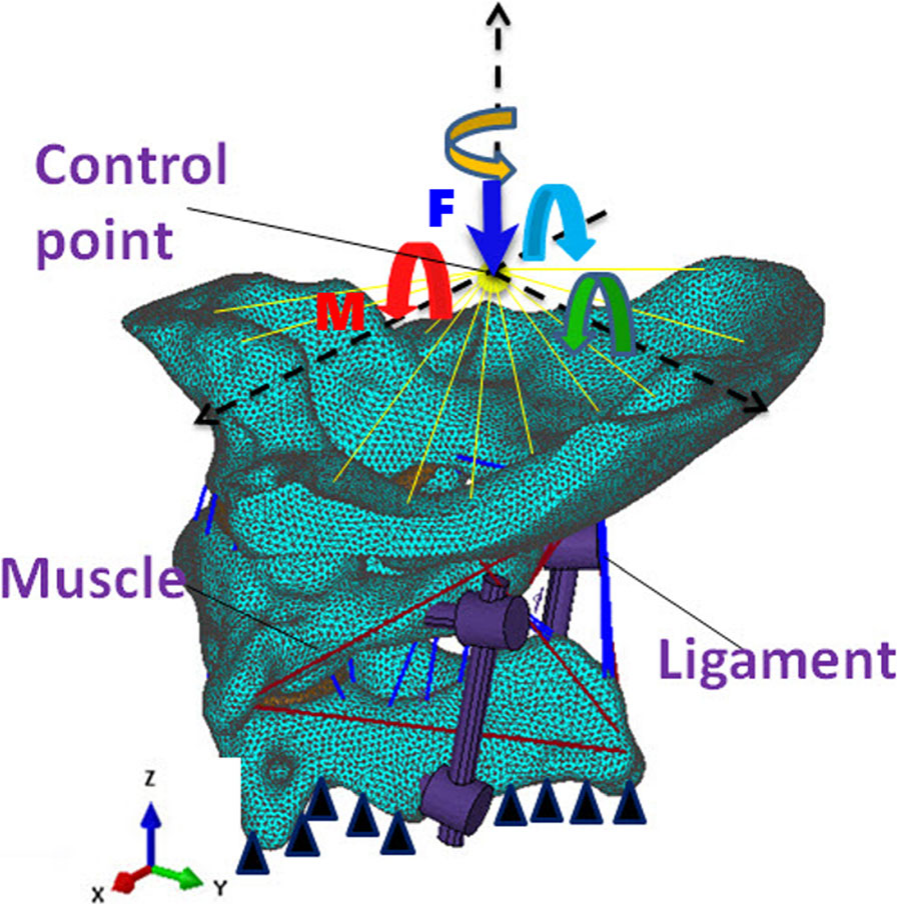
Loading of the finite element models

As the stiffness of implant is much larger than the human bone tissue, embedding constraints were used between the inserted part of the screw and the surrounding bone. The interactions among the vertebral body, cartilage, screw, and rod were defined as binding constraints. The lower surface of the axis was completely restrained; a control point was created on the upper surface of the occipital bone and coupled with all the nodes on the top of the occipital bone. A 40-N vertical downward force and a 1.5-N m moment [16] were applied on the control point along the directions of *X*, *−X*, *Y*, and *Z* axes, respectively, to simulate flexion, extension, lateral bending, and axial rotation of the atlantoaxial joint, as shown in Fig. 3.

## Results

### Three-dimensional angular ROM of the constructs

All constructs significantly reduced ROM at operative levels compared with the intact model. In comparison with the C1PS + C2PS model, the C1PAS + C2PS model increased the ROM by 4.7% and 23.1% in flexion and extension, respectively, and reduced the ROM by 29.9% and 15.8% in lateral bending and axial rotation, respectively; the ClPS + C2LS model reduced the ROM by 3.7%, 26.4%, and 29.6% in flexion, lateral bending, and axial rotation, respectively, and increased the ROM by 28.6% in extension. Segmental ROMs of each model are summarized in Table 3.

**Table 3:** Segmental ROM of each group under different loading conditions

		Flexion (°)	Extension (°)	Lateral bending (°)	Axial rotation (°)
Intact	C0-1	8.34	8.69	3.61	4.7
	C1-2	11.2	7.33	5.17	28.34
	C0-2	19.54	16.02	8.78	33.04
C1PS + C2PS	C0-1	4.3	3.77	3.4	5.4
	C1-2	0.6	0	1.1	0.6
	C0-2	4.87	3.77	4.35	5.82
ClPAS + C2PS	C0-1	5.1	4.4	2.8	4.6
	C1-2	0	0.2	0.3	0.4
	C0-2	5.1	4.64	3.05	4.9
ClPS + C2LS	C0-1	4.49	3.8	3.1	3.9
	C1-2	0.2	0.3	0.2	0.3
	C0-2	4.69	3.85	3.2	4.1

### The stress distribution on the implants

**Fig. 4:**
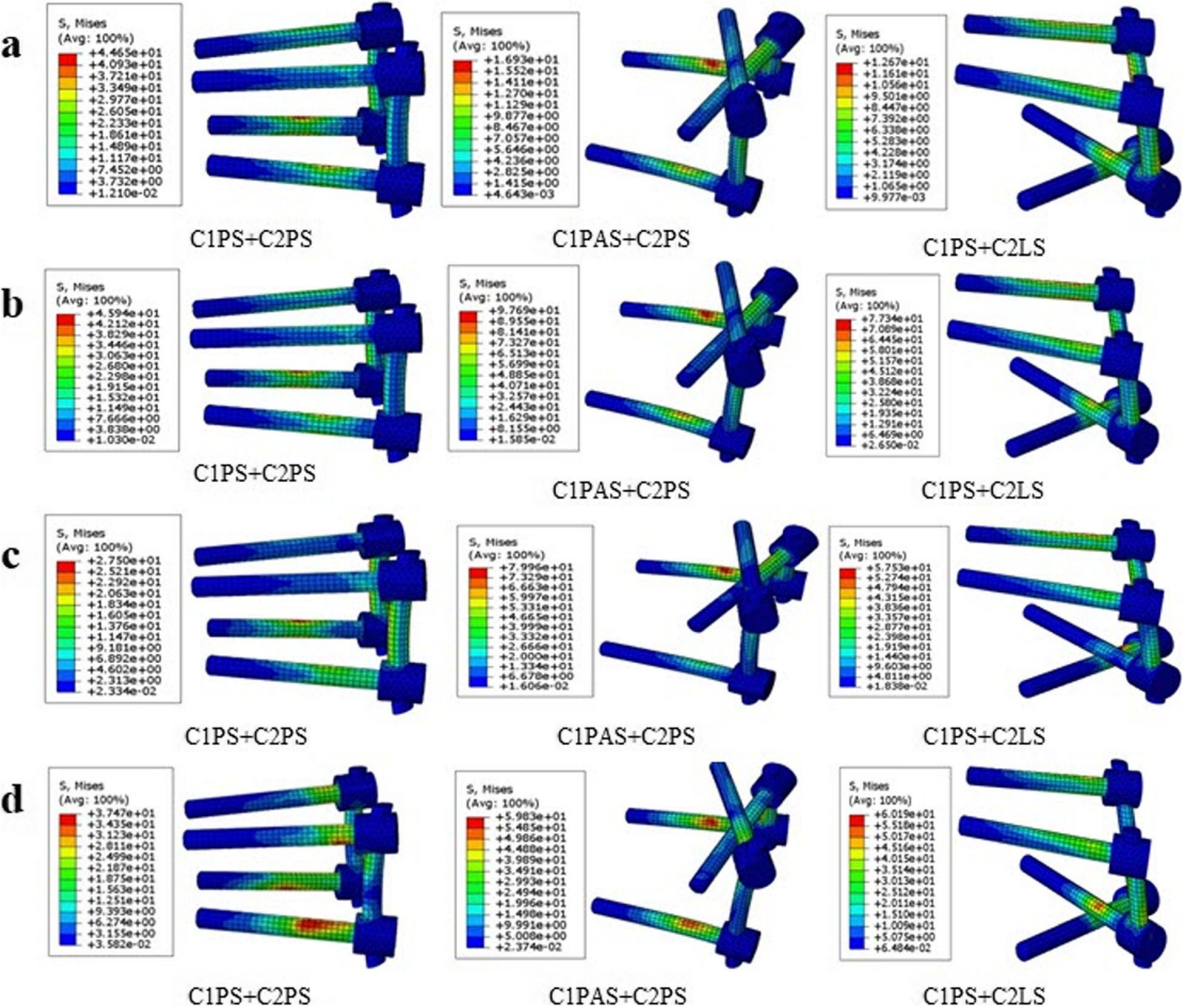
Stress distribution nephograms of implants for the three fixation models when tested in **a** flexion, **b** extension, **c** lateral bending, and **d** axial rotation after applying a 40-N vertical downward force and a 1.5-N m moment

The von Mises stress contour plot (Fig. 3) showed the similar stress distributions of three constructs in the state of equilibrium under the different loading conditions. Implants of all constructs had the maximum stress in extension among the four loading conditions; stress peaks of C1PS + C2PS, ClPAS + C2PS, and ClPS + C2LS models were 45.94 MPa, 97.69 MPa, and 77.34 MPa, respectively (Fig. 4). The ClPAS + C2PS model generated the highest stress under all loading conditions except in flexion. The C1PS + C2PS group showed lower stress levels in all loading conditions, although the peak stress was higher than the other two configurations in flexion.

### Comparison of maximum stress of vertebral bodies

**Fig. 5:**
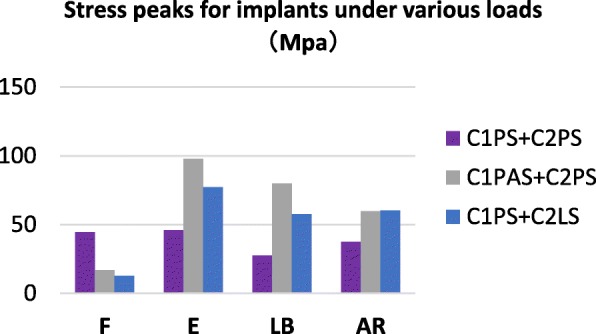
Comparison of stress peaks for implants

The maximum stresses of vertebral bodies mainly occurred at the interaction between the screw and the bone. Figure 5 shows that the C1PS + C2PS model had the higher peak stress in flexion and extension and lower peak stress in lateral bending and axial rotation than that of the other two models. The ClPAS + C2PS model still generated the higher peak stress under all loading conditions except in flexion.

## Discussion

For many years, clinical and experimental studies have indicated that pedicle screw fixation performs well in biomechanical stability. However, the variations of the atlas or axis always result in difficulty in implanting pedicle screws. Fixation techniques of atlas posterior arch screw and axis laminar screw are gradually becoming important supplement methods. Some literature reported that these two fixation techniques can provide comparable stability compared with pedicle screws for the unstable atlantoaxial vertebrae [12, 13]. Few published papers have discussed the different biomechanical effects of the three methods and provided amply illustrated reports. In the background of the rapid development of digital medicine, in-depth biomechanical studies based on numerical simulation can effectively supplement informative data that cannot be obtained through short-term clinical monitoring. This research investigated the mechanism of screw-rod fixation system for atlantoaxial instability, employing classical three-column theory of the spine. The findings can provide theoretical support for the study of atlantoaxial fixation and guide the selection of clinical surgery.

**Fig. 6:**
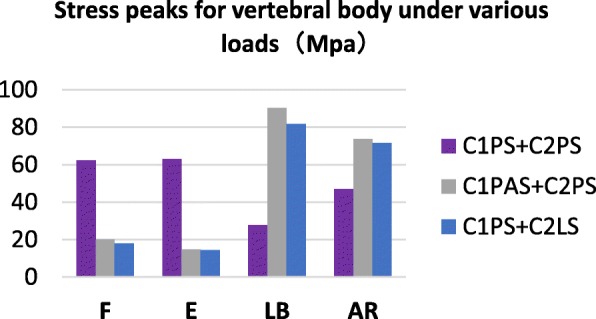
Comparison of stress peaks for vertebral bodies

Guo-Xin et al. [10] studied the feasibility and stability of C1 posterior arch crossing screw and concluded that the PAS rod-screw systems could significantly reduce flexibility in flexion, extension, and rotation compared with the intact model. Wright [5] first reported C2 laminar screw technique, which was shown to provide equivalent acute stability to C2 pars screws by biomechanical analysis. Benke et al. [17] recently reported biomechanical results comparing pedicle and lamina screws in C2-6 constructs and found laminar screws in C2-6 constructs were equivalent to transpedicular fixation in flexion-extension and were significantly more rigid in axial rotation and less rigid in lateral bending than pedicle screws. However, these authors did not give some theoretical analysis about the subtle differences among these techniques. Our finding suggests that the ClPAS + C2PS and ClPS + C2LS models may offer equivalent stability to the C1PS + C2PS model in flexion, lower stability in extension, and higher stability in lateral bending and axial rotation. Fixation stability mainly depends on the anchoring range and kinematics of atlantoaxial vertebra. For C1PS + C2PS fixation, four screws inserted through the posterior part of the vertebral body to the front form a three-column fixation; for ClPAS and C2LS, anchoring area of the screws did not arrive in the anterior column, and that may explain why the ClPAS + C2PS and ClPS + C2LS models offered lower stability in extension (Fig. 6). Besides, unlike the C1PS + C2PS fixation, the ClPAS and C2LS techniques belonged asymmetrical cross fixation, which may have effects on resistance to lateral bending and axial rotation.

**Fig. 7:**
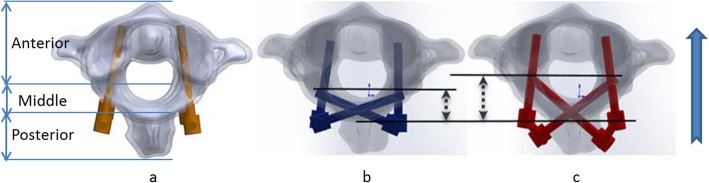
Comparison of anchoring range for **a** PS, **b** C1PAS, and **c** C2LS

Stress distribution on the implants is closely related to the long-term stability of fixation techniques. But much work has not been focused on investigating it. Middle and posterior portions of the screws were stress-concentrated areas. This was because the screws required to transmit the mechanical load to the rear rods. When compared with the C1PS + C2PS fixation, the ClPAS + C2PS and ClPS + C2LS showed high stress peaks on implants in extension, lateral bending, and axial rotation consistently, which indicated asymmetric fixations may result in high stress on the implants. And it demonstrates that symmetrical internal fixation can effectively improve the stress distribution of implanted segments under normal physiological loads and provide a stable force environment for the implants. Compared with ClPAS + C2PS, C1PS + C2LS showed lower stress peak in flexion, extension, and lateral bending, with similar value in axial rotation. Based on the classical three-column fixation theorem [18], the anchoring length of C1 posterior arch screw is shorter than that of the C2 lamina screw along the direction from the posterior column to the anterior column (Fig. 7). Load transmission among atlantoaxial complex can spread to the anterior, middle, and posterior columns, in flexion, extension, and lateral bending. However, atlantoaxial mechanical mechanism does not follow the three-column theorem in axial rotation. Therefore, ClPAS + C2PS and ClPS + C2LS groups had the same stress peak on implants in axial rotation. The peak stress of the vertebra generally appeared along the adjacent area between the bone and screws. The C1PS + C2PS fixation led to high stress in flexion-extension. It could be that the three-column fixation caused strong interaction since its strong biomechanical strength in anchoring along flexion-extension direction (Fig. 7). The ClPAS + C2PS and ClPS + C2LS fixations led to high stress in lateral bending and axial rotation, which reflected the crossing screw fixation provided better effect on anti-bending and anti-rotation from the side.

## Conclusions

From the mechanical viewpoint, the ClPAS + C2PS and ClPS + C2LS fixations may offer higher stability to ClPS + C2PS in lateral bending and axial rotation, but the ClPS + C2PS can put the implants in a position of mechanical advantage.

## Data Availability

Corresponding author CL can be contacted to request the raw data.

## Abbreviations

AL: Alar ligament
ALL: Anterior longitudinal ligament
C0: Lower part of the occipital
C0-1: Lower part of the occipital and atlas
C1: Atlas
C1-2: Atlas and axis
C1PS: C1 pedicle screw
C2: Axis
C2LS: C2 lamina screw
C2PS: C2 pedicle screw
CL: Capsular ligament
ClPAS: C1 posterior arch screw
FEM: Finite element model
LAD: Apical ligament of dens
LF: Flavum ligament
MT: Membranae tectoria
PLL: Posterior longitudinal ligament
RCP: Rectus capitis posterior
ROM: Range of motion
TL: Transverse ligament
